# Efficacy and Safety of Teprotumumab in Patients With Thyroid Eye Disease of Long Duration and Low Disease Activity

**DOI:** 10.1210/clinem/dgad637

**Published:** 2023-10-31

**Authors:** Raymond S Douglas, Steven Couch, Sara T Wester, Brian T Fowler, Catherine Y Liu, Prem S Subramanian, Rosa Tang, Quang T Nguyen, Robi N Maamari, Shoaib Ugradar, Kate Hsu, Michael Karon, Marius N Stan

**Affiliations:** Department of Ophthalmology, Cedars Sinai Medical Center, Los Angeles, CA 90048, USA; John F. Hardesty MD Department of Ophthalmology and Visual Sciences, Washington University School of Medicine, St Louis, MO 63130, USA; Bascom Palmer Eye Institute, University of Miami, Miami, FL 33136, USA; Department of Ophthalmology, The University of Tennessee Health Science Center, Hamilton Eye Institute, Memphis, TN 38163, USA; Department of Ophthalmology, Shiley Eye Institute, University of California San Diego, La Jolla, CA 92037, USA; Departments of Ophthalmology, Neurology, and Neurosurgery, UC Health Sue Anschutz-Rodgers Eye Center, University of Colorado School of Medicine, Aurora, CO 80045, USA; Department of Surgery, Division of Ophthalmology, Uniformed Services University of the Health Sciences, Bethesda, MD 20814, USA; Eye Wellness Center, Neuro-Eye Clinical Trials, Inc., Houston, TX 77074, USA; Department of Endocrinology, Touro University, Henderson, NV 89014, USA; John F. Hardesty MD Department of Ophthalmology and Visual Sciences, Washington University School of Medicine, St Louis, MO 63130, USA; Department of Ophthalmology, Cedars Sinai Medical Center, Los Angeles, CA 90048, USA; Clinical Development, Horizon Therapeutics plc, Deerfield, IL 60015, USA; Clinical Development, Horizon Therapeutics plc, Deerfield, IL 60015, USA; Division of Endocrinology, Diabetes and Metabolism, Mayo Clinic, Rochester, MN 55905, USA

**Keywords:** thyroid eye disease, teprotumumab, Graves disease, inactive, chronic

## Abstract

**Context:**

Early inflammatory thyroid eye disease (TED) can lead to symptomatic chronic disease, including disabling proptosis. Teprotumumab, an insulin-like growth factor-1 receptor (IGF-1R) inhibitor, previously demonstrated efficacy in acute, high-inflammation TED trials.

**Objective:**

We present data from the first placebo-controlled trial with teprotumumab in chronic/low disease activity TED.

**Methods:**

This randomized double-masked, placebo-controlled trial, conducted at 11 US centers, enrolled adult participants with TED duration of 2 to 10 years, Clinical Activity Score (CAS) ≤ 1 or no additional inflammation or progression in proptosis/diplopia for ≥1 year, proptosis ≥3 mm from before TED and/or from normal, euthyroid/mildly hypo/hyperthyroid, no prior teprotumumab, and no steroids within 3 weeks of baseline. Patients received (2:1) intravenous teprotumumab or placebo once every 3 weeks (total 8 infusions). The primary endpoint was proptosis (mm) improvement at Week 24. Adverse events (AEs) were assessed.

**Results:**

A total of 62 (42 teprotumumab and 20 placebo) patients were randomized. At Week 24, least squares mean (SE) proptosis improvement was greater with teprotumumab (−2.41 [0.228]) than with placebo (−0.92 [0.323]), difference −1.48 (95% CI −2.28, −0.69; *P* = .0004). Proportions of patients with AEs were similar between groups. Hyperglycemia was reported in 6 (15%) vs 2 (10%) and hearing impairment in 9 (22%) vs 2 (10%) with teprotumumab and placebo, respectively. AEs led to discontinuation in 1 teprotumumab (left ear conductive hearing loss with congenital anomaly) and 1 placebo patient (infusion-related). There were no deaths.

**Conclusion:**

Teprotumumab significantly improved proptosis vs placebo in longstanding/low inflammation TED, demonstrating efficacy regardless of disease duration/activity. The safety profile was comparable to that previously reported.

Thyroid eye disease (TED), also known as Graves orbitopathy, is an autoimmune disease manifesting retro-orbital fat and muscle inflammation and tissue expansion with progressive signs and symptoms of strabismus, diplopia, and proptosis, resulting in significant disability and morbidity (1). Historically, it has been described as a biphasic disease with initial acute or dynamic inflammation of orbital tissues (pain, redness, edema) that crescendos followed by a diminution and stabilization of inflammation with continued tissue expansion and fibrosis often referred to as the chronic phase. This characterization is simplistic, as not all patients demonstrate initial acute inflammatory signs and there can be negligible reduction in the signs and symptoms over time due to continued tissue expansion (2). Today, evidence indicates that TED should be viewed more as a chronic long-term symptomatic disease in which patients remain highly symptomatic and rarely return to their premorbid state (2-5).

Data on medical treatment for the chronic, long-term symptoms are lacking. For example, the European Group on Graves’ Orbitopathy (EUGOGO) 2021 guidelines recommended intravenous glucocorticoids in combination with mycophenolate as first-line treatment but limited data exist on their effectiveness, particularly on the progressive outcomes of proptosis and diplopia in chronic TED where inflammation is low (6-8). Data suggest that conventional pharmacotherapies do not help in chronic TED, and disease manifestations have typically been treated surgically.

Teprotumumab, an insulin-like growth factor-1 receptor (IGF-1R) inhibitor, was the first drug approved for TED treatment, in 2020 in the United States, based on 2 randomized, double-masked, placebo-controlled clinical trials (9, 10). Patients with active TED (determined by a Clinical Activity Score (CAS) ≥ 4) (11) and onset within 9 months were included in these trials. In the open-label extension from one of these studies, placebo patients with a mean disease duration of approximately 13 months were treated with teprotumumab (12). Based on these studies, a 2022 European Thyroid Association and American Thyroid Association consensus statement recommended the first-line use of teprotumumab (where available) for a wide group of patients with moderate to severe TED presenting with active inflammation and significant proptosis and/or diplopia (13).

Although several observational reports have been published indicating favorable effects of teprotumumab in a wide group of patients with chronic TED, there are no well-controlled study data with teprotumumab in patients with longer duration of TED without active inflammation (14). We report results of the first randomized, double-masked, placebo-controlled trial in patients with TED with low clinical activity. The objective of this study was to investigate the efficacy, safety, and tolerability of teprotumumab in patients with long-duration TED and low disease activity as assessed by the CAS.

## Methods

### Study Design

The trial is a randomized, double-masked, placebo-controlled, parallel-group, multicenter, phase 4 trial, conducted at 11 sites in the United States. Patients were screened within 4 weeks prior to the Baseline Visit.

The study comprised a 24-week masked period. Patients were randomized in a 2:1 ratio without stratification to receive 8 infusions of teprotumumab (10 mg/kg for the first infusion and 20 mg/kg for the remaining 7 infusions) or placebo once every 3 weeks. Infusions were scheduled on Day 1 (baseline) and Weeks 3, 6, 9, 12, 15, 18, and 21 (with a final visit at Week 24) (Supplemental Figure S1 (15)).

A randomization schedule was performed by the contract research organization using the electronic data capture (EDC) system. The patients, investigators, trial site personnel (excluding the formulating pharmacists) and data assessors were masked to assigned treatment until study end.

Unless there were infusion-associated events, the first 2 infusions of the double-masked periods were to be administered over 90 minutes, but not less than 80 minutes, and subsequent infusions over approximately 60 minutes but not less than 50 minutes. Patients were monitored for adverse events (AEs) during and for 60 minutes post infusion for the first 3 infusions, and for up to 30 minutes after subsequent infusions.

At the end of the double-masked treatment period (Week 24), teprotumumab or placebo patients who were nonresponders (< 2-mm reduction in proptosis in the study eye) could enter an open-label period and receive 8 infusions of teprotumumab once every 3 weeks. Results from the masked period are reported here. The trial was approved by the U.S. Food and Drug Administration (FDA) and by the institutional review board or independent ethics committee at each trial site. The trial was conducted in accordance with the International Conference on Harmonization Good Clinical Practice guidelines and the principles of the Declaration of Helsinki. All patients provided written informed consent.

Horizon Therapeutics sponsored the trial. All authors made the decision to submit the manuscript for publication.

### Study Population

Key inclusion criteria were age ≥18 years, with a TED diagnosis of at least 2 years but < 10 years before screening and a diagnosis of stable, chronic (inactive) TED. Stable/inactive disease was defined as a CAS ≤ 1 in both eyes prior to screening for at least 1 year or all of the following for at least 1 year before screening: no proptosis progression, no diplopia progression in patients with history of diplopia, and no new inflammatory TED symptoms. Patients must have had ≥ 3-mm increase in proptosis from before diagnosis of TED and/or proptosis ≥ 3 mm above normal values for race and sex. Patients were required not to be pregnant, and to be euthyroid although mild (free thyroxine and triiodothyronine levels <50% +/− normal limits) hypothyroidism or hyperthyroidism was allowed at screening. Hemoglobin A1C (Hb1Ac) levels were required to be ≤ 8% at baseline, and those with a history of inflammatory bowel disease, ulcerative colitis, or Crohn's disease must have been in remission for ≥ 3 months with no bowel surgery in the 6 preceding months.

Key exclusion criteria were previous strabismus surgery, orbital radiation, or orbital decompression in the study eye, decreasing visual acuity due to optic neuropathy or a visual field or color vision defect secondary to optic nerve involvement in the previous 6 months, any glucocorticoid use within 3 weeks before screening, rituximab use in the previous 12 months, tocilizumab use in the previous 6 months, any nonsteroid immunosuppressive agent in the previous 3 months before infusion, or any monoclonal antibody in the 3 previous months before screening, or any previous treatment with teprotumumab. A complete list of inclusion and exclusion criteria is provided in Supplemental Table S1 (15). The use of certain medications was restricted (Supplemental Table S2 (15)), and exclusion criteria included specific preexisting medical conditions. Patients needed to meet criteria at both screening and baseline.

### Outcomes and Assessments

The primary outcome was the change of proptosis measurements (mm) in the study eye from baseline at Week 24. Other outcomes, all at Week 24, were proptosis responder rate (percentage of patients with ≥ 2 mm reduction from baseline in the study eye without deterioration [≥ 2 mm increase] in the fellow eye), change from baseline in the Graves’ Ophthalmopathy Quality of Life (GO-QOL) questionnaire appearance and visual functioning subscales, change from baseline in diplopia as ordinal response categories, binocular diplopia responder rate (percentage of patients with baseline diplopia >0 who had reduction ≥ 1 grade, using the Bahn-Gorman scale), complete binocular diplopia responder rate (percentage of patients with baseline diplopia > 0 and score of 0 at Week 24). Change from baseline at Week 24 in diplopia as ordinal response categories was based on all randomized patients in the trial. Patients with/without diplopia at baseline were assessed for improvement (by 1 grade), significant improvement (2 or 3 grades), no change, worsening (1 grade) or significant worsening (2 or 3 grades) at Week 24. A schedule of assessments is provided (Supplemental Table S3 (15)). An exploratory outcome assessed the effect of teprotumumab on change from baseline at Week 24 in retro-orbital muscle volumes for inferior rectus, superior rectus, medial rectus, lateral rectus, and volume of orbital fat (measured by magnetic resonance imaging [MRI]) on patients in whom MRI was obtained from one study site (R.S.D.). Three-dimensional volumetric analyses of the orbital fat and muscle volume were performed using the previously validated 3D image analysis software, MIMICS (Materialise, Leuven, Belgium) (16). Muscle and fat volume measurements were performed using a previously described technique (17). Quasicoronal images were rotated as necessary to align the midline of the brain to vertical and eradicate potential for errors from head tilting during the scan. Using the software, orbital fat and muscle tissue were defined manually. Fat was measured between the septum and the orbital outlet of the optic canal. A mask was created by manual segmentation, slice by slice to generate a 3D model through voxel addition and expressed in millimeters cubed. Segmentation and measurements were performed independently by 2 masked graders.

Safety assessments included vital signs, adverse events (AE) and AE of special interest (AESI, infusion reactions, hyperglycemia, hearing impairment, new onset inflammatory bowel disease and exacerbation of inflammatory bowel disease). Hematology, fasting chemistry, thyroid function tests and HbA1c tests were conducted during study visits by a central laboratory (Supplemental Table S3 (15)).

### Statistical Analysis

The sample size was determined assuming that the mean difference in proptosis change between the groups is at least 2.0 mm (clinically relevant difference) with a standard deviation of proptosis change values of 2.5 for both groups (larger than observed in Phase 2 and Phase 3 active TED trials). A total of 57 patients (38 in the teprotumumab, and 19 in the placebo group) was planned to detect at least a 2-mm mean difference between the treatment groups in the change from baseline of proptosis values at Week 24, in order to have 81% power at the 2-sided .05 level of significance. Efficacy endpoints were sequentially tested in a prespecified order and were significant only if statistical significance was achieved for the primary efficacy endpoint, and each preceding endpoint at the .05 significance level.

The primary analysis dataset was the intention-to-treat (ITT) analysis set (all randomized patients). Supplementary analyses were also conducted in the modified intention-to-treat (mITT) analysis set who received ≥ 1 dose of study drug and had ≥ 1 post-baseline measurement of the primary efficacy endpoint and per protocol analysis set, which included randomized patients who received ≥ 1 dose of drug during the masked period, had ≥ 1 post-baseline measurement, completed the masked period, without major protocol violations that would challenge data validity. The safety analysis set included patients who had received ≥ 1 dose of study drug. Missing data was not imputed unless methods for handling missing data are specified. For categorical endpoints, patients who were missing Week 24 evaluation were considered nonresponders.

### Statistical Tests

A mixed model for repeated measures (MMRM) analysis of covariance model fitting to the individual change from baseline scores for the study eye was used to analyze change from baseline in proptosis and included baseline value, treatment group, visit, visit-by-treatment, and visit-by-baseline value as fixed effects, and patients as a random effect. The change from baseline at Week 24 in GO-QOL overall and subscale scores were analyzed using MMRM. Proptosis responder rate at Week 24 for study eye, diplopia responder rate at Week 24, and complete diplopia responder rate at Week 24 were analyzed using the Fisher exact test. The change from baseline at Week 24 in diplopia as ordinal response categories was analyzed by the proportional odds model (18) which considers diplopia status (worsened, improved, or no change) for all randomized patients with an odds ratio calculated for patients treated with teprotumumab vs placebo.

The trial is registered with Clinicaltrial.gov: NCT04583735 (Enrollment closed).

### Role of the Funding Source

The study sponsor, Horizon Therapeutics, had a role in designing the study, collecting data, analysis and interpretation of data, and writing the final report. The corresponding author had full access to the data and final responsibility for the decision to submit for publication.

## Results

### Characteristics of the Study Population

**Figure 1.**
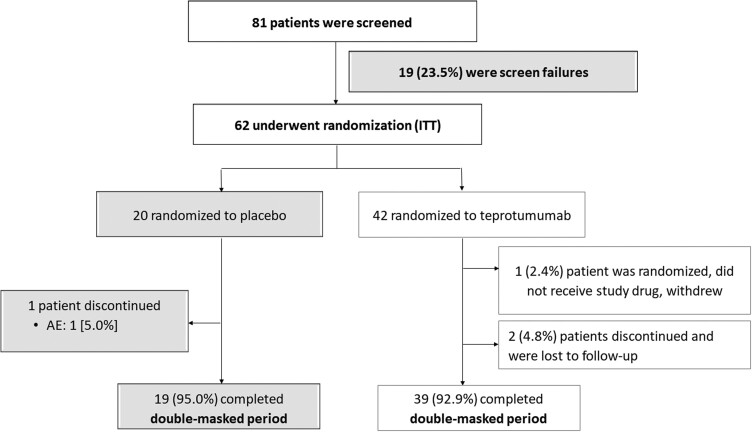
Enrollment, randomization, and completion of treatment period. Patients not meeting criteria at Day 1 were screening failed. Abbreviations: AE, adverse event, ITT, intention-to-treat population.

Eighty-one patients were screened between August 12, 2021, and September 15, 2022, and 62 were randomized (42 to teprotumumab and 20 to placebo [ITT population]). Thirty-nine of 42 patients (93%) in the teprotumumab group, and 19/20 (95%) in the placebo group completed the double-masked treatment period (Fig. 1).

Demographics and disease characteristics were predominantly balanced across groups at baseline (Table 1), although a higher proportion of patients were female in the placebo arm. Mean (SD) age overall was 48.7 (14.9) years.

**Table 1.** Demographics and disease characteristics at baseline

	Placebo (N = 20)	Teprotumumab (N = 42)
**Age (years), mean (SD)**	49.0 (16.5)	48.6 (14.4)
**Sex, n (%)**		
Male	2 (10.0)	10 (23.8)
Female	18 (90.0)	32 (76.2)
**Race, n (%)**		
White	12 (60.0)	22 (52.4)
Black or African American	5 (25.0)	10 (23.8)
Asian	1 (5.0)	7 (16.7)
Other	2 (10.0)	3 (7.1)
**Years since diagnosis of thyroid**		
**eye disease**		
Mean (SD)	5.4 (1.61)	5.1 (1.88)
Median (range)	5.8 (2.7-7.8)	4.9 (2.2-8.7)
Thyroxine, free (pmol/L), mean (SD)	19.04 (4.378)	17.86 (4.273)
Triiodothyronine, free (pmol/L), mean (SD)	4.48 (0.973)	4.38 (0.883)
Thyrotropin (mIU/L), Mean (SD)	1.78 (1.890)	4.08 (7.838)
**Autoimmune diseases**		
Autoimmune thyroiditis	0	2
Type 1 diabetes	0	1
Rheumatoid arthritis	1	2
**Tobacco use history, n (%)**		
Former	6 (30.0)	11 (26.2)
Current	2 (10.0)	6 (14.3)
Never	12 (60.0)	25 (59.5)
**Proptosis (mm) for study eye, mean (SD)**	24.0 (2.82)	24.6 (3.01)
**CAS in study eye, mean (SD)**	0.5 (0.51)	0.3 (0.47)
**Patients with diplopia, n (%)**	4	14
Intermittent diplopia	2 (10.0)	6 (14.3)
Inconstant diplopia	1 (5.0)	2 (4.8)
Constant diplopia	1 (5.0)	6 (14.3)
**GO-QOL transformed score, mean (SD)**		
Overall (total score)	60.4 (20.32)	66.3 (21.73)
Visual functioning subscale	81.4 (18.84)	86.4 (20.03)
Appearance subscale	40.0 (28.56)	46.4 (29.55)

Graves’ ophthalmopathy quality of life questionnaire (GO-QOL): range 0-100, with higher scores indicating better quality of life. Levels of thyroxine, thyrotropin, triiodothyronine, and autoimmune diseases were assessed in the safety population. Abbreviation: CAS, Clinical Activity Score.

### Outcomes

**Figure 2.**
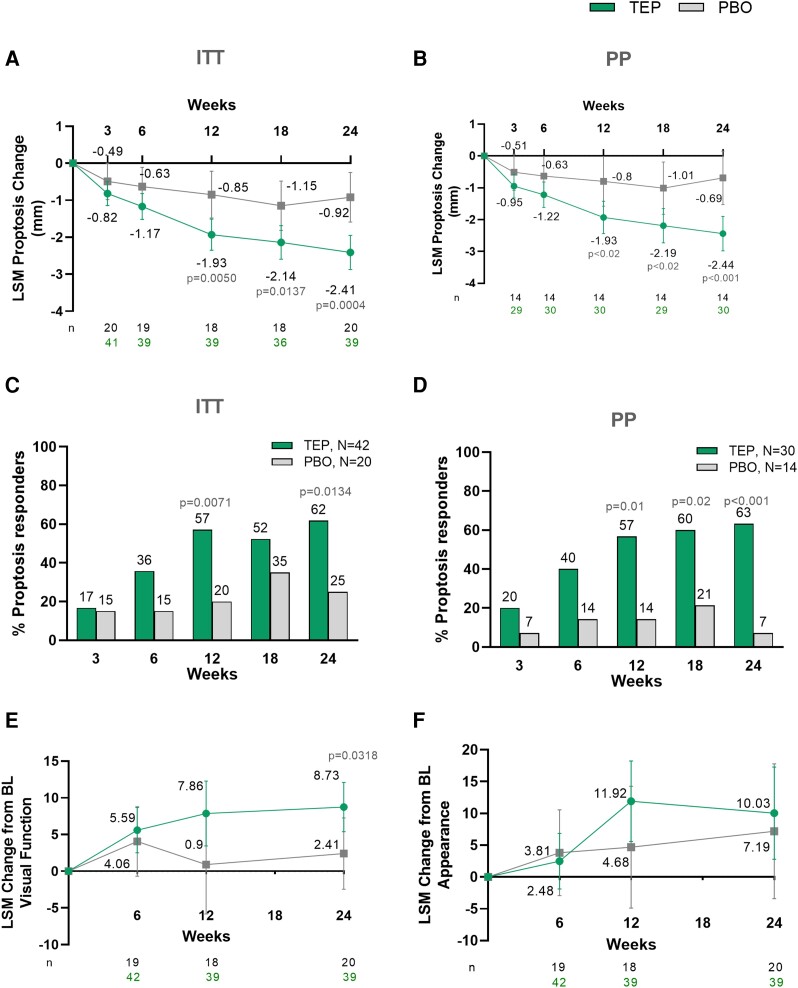
A, LS Mean change from baseline in proptosis in mm (primary endpoint, ITT, MMRM). B, LS Mean change from baseline in proptosis in mm (per protocol, MMRM). C, percentage of patients with a proptosis response (ITT). D, percentage of patients with a proptosis response (per protocol). LS Mean change from baseline in the (E) GO-QOL Visual Function subscale and (F) GO-QOL Appearance-related subscale (MMRM). Error bars represent 95% CI. Abbreviations: AP, appearance subscale; BL, baseline; GO-QOL, Graves’ Ophthalmopathy Quality of Life questionnaire; LS, least squares; MMRM, mixed model for repeated measures, VF, visual function subscale.

The primary endpoint was met. At Week 24, in the ITT population, least squares (LS) mean (SE) change in proptosis from baseline in teprotumumab group was −2.41 (0.23) mm vs −0.92 (0.32) mm in the placebo group (between-group difference, −1.48 mm; 95% CI −2.28, −0.69; *P* = .0004), was greater at all time points and significantly greater than placebo at Weeks 12, 18, and 24 (Fig. 2A). A significantly greater proportion of patients, 26 of 42 (61.9%), in the teprotumumab group had a proptosis response vs those receiving placebo (5 of 20, 25%); treatment difference 36.9% (95% CI 5.4, 59.2; *P* = .0134) (Fig. 2C). In the per protocol analysis, results were consistent (Fig. 2B and 2D).

Significantly greater improvements from baseline were observed with teprotumumab vs placebo at Week 24 in the GO-QOL Visual Function subscale: LS mean (SE) 8.73 (1.661) vs 2.41 (2.329), respectively, with treatment difference 6.31 (95% CI 0.57, 12.06, *P* = .0318) (Fig. 2E). On the GO-QOL Appearance-related subscale, an improvement from baseline of 10.03 (3.592) was observed in the teprotumumab group vs 7.19 (5.069) in placebo, which was not statistically significant: treatment difference 2.85 (95% CI −9.62, 15.32, *P* = .649) (Fig. 2F).

A small number of enrolled patients had diplopia at baseline and the proportions were unevenly matched in the teprotumumab (14/42, 33%) and placebo arms (4/20, 20%). Improvements in diplopia were not significant for teprotumumab vs placebo: Of patients with diplopia at baseline, 6/14 (42.9%) patients in the teprotumumab group had an improvement of ≥ 1 grade in diplopia, vs 2/4 (50%) patients in the placebo group. Four of 14 (28.6%) in the teprotumumab group vs 1/4 (25%) in the placebo group had complete resolution of diplopia at Week 24. Using the proportional odds model with diplopia reported as ordinal response categories, the odds ratio for change from baseline at Week 24 favored teprotumumab but was not significant (2.13; 95% CI 0.39, 11.60; *P* = .3815).

**Figure 3.**
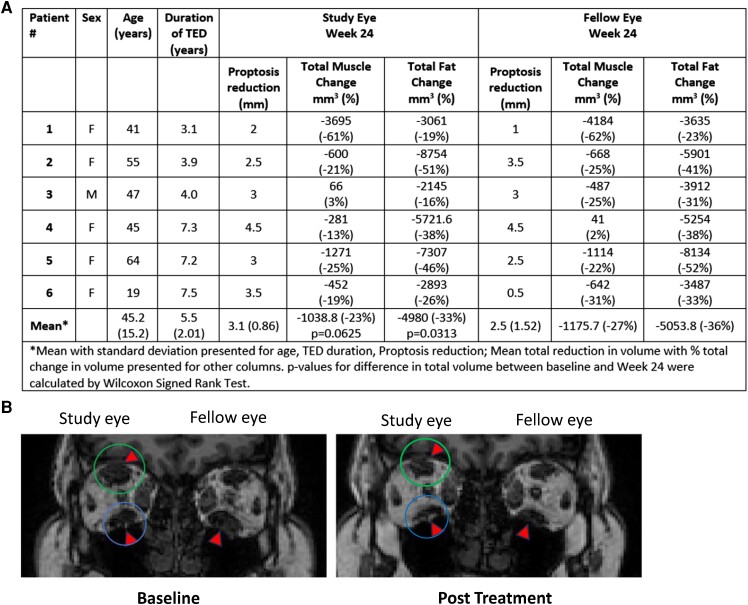
A, Patient characteristics and reduction in orbital muscle and fat volume in 6 patients (study eye and fellow eye) in the teprotumumab group. B, MRI at baseline and Week 24 in an individual patient (#5 in panel A) treated with teprotumumab. The green circle indicates the superior rectus and the blue circle indicates the inferior rectus of the study eye. The patient was female, aged 64 with approximately 7 years since diagnosis of TED and no diplopia at baseline. At Week 24, proptosis was reduced in the study eye and fellow eye by 3 and 2.5 mm, respectively and GO-QOL improved by 49 (overall), 75 (appearance), and 23 (visual function).

**Figure 4.**
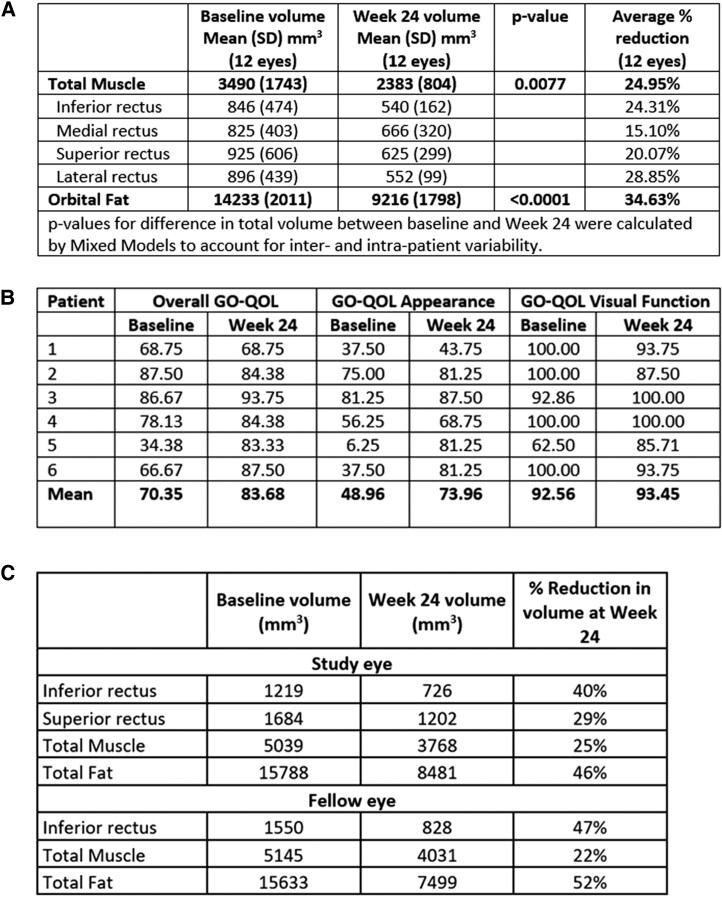
A, Reduction in volume of each orbital muscle and fat for 6 patients (12 eyes) with MRI imaging at baseline and Week 24. B, GO-QOL overall, appearance, and visual function scores in the 6 patients with MRI imaging. C, muscle and fat volumes at baseline and Week 24 in an individual patient (#5 in Fig. 3A and Fig. 4B, imaged in 3B) treated with teprotumumab.

For 6 patients (12 eyes) in the teprotumumab group who had orbital MRI at baseline and Week 24, an improvement in proptosis was accompanied by a mean decrease from baseline in retro-orbital total muscle volume of 24.95%, with mean volume decrease of 24.31% in the inferior rectus, 20.07% in the superior rectus, 15.1% in the medial rectus, and 28.85% in the lateral rectus. A mean decrease in orbital fat volume of 34.63% was observed (Fig. 3, Figure 4A). The average overall GO-QOL and appearance scores improved by approximately 13 and 25 points, respectively, while the visual function score, which was high at baseline, was not further improved (Fig. 4B). Muscle and fat volume changes for the individual patients are presented in Fig. 4C.

### Safety

Adverse events (AEs) were reported in similar proportions of patients in the teprotumumab (33 of 41, 80.5%) and placebo (16 of 20, 80%) groups. The AEs described in previous teprotumumab studies and as included in the prescribed label for teprotumumab are presented in Table 2 and those reported in at least 2 patients are reported in Supplemental Table S4 (15). The most frequently reported AE in the teprotumumab group was muscle spasms (41.5%) vs 10% in the placebo group. Spasms occurred most frequently in the lower extremities; all were Grade 1, of mild intensity, and none were serious or led to treatment discontinuation. Other AEs reported more frequently in the teprotumumab vs placebo group (≥ 5.0% difference) were fatigue (22.0% vs 10.0%), headache (17.1% vs 10.0%), dry skin (12.2% vs 0%), eye pain (12.2% vs 5.0%), eye pruritus (7.3% vs 0%), Hb1Ac increased (7.3% vs 0%) and hypertension (7.3% vs 0%). These events were of mild to moderate intensity, none were serious or resulted in discontinuation.

**Table 2.** Patients with treatment-emergent adverse events during the double-masked treatment period

	Teprotumumab (N = 41) n (%)	Placebo (N = 20)*^a^* n (%)
Any adverse events	33 (80.5)	16 (80.0)
Serious adverse events*^b^*	1 (2.4)	1 (5.0)
Adverse events leading to study drug discontinuation*^c^*	1 (2.4)	1 (5.0)
Adverse events leading to death	0	0
Adverse events of special interest	15 (36.6)	7 (35.0)
Infusion reaction	2 (4.9)	3 (15.0)
Hyperglycemia	6 (14.6)	2 (10.0)
Hearing impairment	9 (22.0)	2 (10.0)
New onset/exacerbation of bowel disease	0	0
Other adverse events of importance		
Muscle spasms	17 (41.5)	2 (10.0)
Nausea	2 (4.9)	1 (5.0)
Alopecia	2 (4.9)	0
Diarrhea	8 (19.5)	4 (20.0)
Fatigue	9 (22.0)	2 (10.0)
Dysgeusia	4 (9.8)	1 (5.0)
Headache	7 (17.1)	2 (10.0)
Dry skin	5 (12.2)	0
Nail bed disorder	2 (4.9)	0

The safety analysis set included patients who had received at least one dose of drug. Within each AESI (adverse event of special interest) category, patients are counted once for each system organ class and once for each preferred term.

^*a*^Includes patient who received one dose of teprotumumab in error.

^*b*^Teprotumumab: conductive hearing loss in patient with congenital anomaly; Placebo: Patient experienced diabetic ketoacidosis after receiving teprotumumab for one infusion (first infusion) in error, despite being randomized (assigned) to the placebo group.

^*c*^Teprotumumab: conductive hearing loss (described above) occurred in double-masked period; patient completed masked period and discontinued in open-label period; Placebo: infusion reaction.

In most patients (97.6% for the teprotumumab, and 95% for the placebo group) AEs were Grade 1 or 2. Two AEs led to discontinuation: one placebo patient (infusion-related) and one teprotumumab patient with left ear conductive hearing loss (patient had a congenital anomaly); the event occurred in the masked period, the patient completed the masked period and discontinued in the open-label period).

AEs of special interest (AESI) were reported in 15 (36.6%) patients in the teprotumumab and 7 (35%) in the placebo group. Hearing impairments were reported in 9 (22%) patients treated with teprotumumab and 2 (10%) who received placebo (Supplementary Table S5 (15)). In the teprotumumab group, 9 patients reported 12 hearing impairments, including 1 autophony, 1 eustachian tube dysfunction, 1 tympanic membrane disorder, 2 tinnitus, 4 hypoacusis, 1 conductive deafness (in a patient with congenital anomaly), 2 deafness unilateral (in the same patient). In the placebo group, 2 patients reported 3 events of tinnitus. In the teprotumumab group, the event of conductive deafness in a patient with congenital anomaly was serious; the patient completed the masked period. All the other events were mild or moderate in severity. Patients experiencing hearing impairment were instructed to contact the study site for evaluation and assessments, which could include an audiogram. At the time of data collection, 3 of the 9 patients had recovered/were recovering, although the duration of follow-up was short.

Hyperglycemic events were reported in 6 (14.6%) patients in the teprotumumab and 2 (10%) in the placebo group (Supplementary Table S6 (15)). Events reported included diabetes in 1 placebo and 2 teprotumumab patients. A serious event of diabetic ketoacidosis (DKA) was reported in 1 placebo-assigned patient. At baseline, the patient had undiagnosed diabetes mellitus and uncontrolled glucose levels and experienced the DKA event after receiving one infusion of teprotumumab in error (first dose). The DKA event resolved with treatment and the patient completed the trial receiving placebo at each subsequent infusion. Other hyperglycemia events in the teprotumumab group were mild and were managed with adjustment of medication with none leading to discontinuation. One patient had a history of diabetes mellitus, and for 4 other patients, Day 1 HbA1c values (range, 5.7%-6.0%) suggest the possibility of preexisting prediabetes/impaired glucose tolerance (prediabetes: HbA1c 5.7%-6.4%, as defined by the American Diabetes Association, 2019; https://diabetes.org/diabetes/a1c/diagnosis).

Infusion-related reactions were reported in 2 (4.9%) teprotumumab and 3 (15%) placebo patients and were mostly managed with medication; 1 patient in the placebo group (5%) discontinued due to an infusion reaction (dull cardiac-related chest pain, pressure-like, nonradiating, slight difficulty breathing).

Serious AEs were reported in 1 (2.4%) teprotumumab (conductive deafness in patient with congenital anomaly) and 1 (5%) placebo-assigned patient (DKA in patient with undiagnosed diabetes mellitus who received teprotumumab infusion in error, described above).

## Discussion

TED activity historically has been assessed based on CAS, which includes the presence of eyelid and conjunctival edema or redness, caruncle swelling, and orbital pain in primary gaze or with eye movement. The presence of 3 or more of these has previously justified classifying patients with active or inflammatory disease (11). Yet patients without these signs and symptoms can remain highly symptomatic with findings such as ocular surface disease, dryness/grittiness, itchy/watery eyes, or blurred/impaired vision, along with the more debilitating and progressive signs/symptoms of proptosis and diplopia (2, 3). Studies of patients with TED have reported impairment related to these symptoms for as long as 10 to 11 years, including in those who had received radiotherapy or steroids at initial presentation (19, 20).

Patients can present with inflammatory or noninflammatory symptoms across the natural history of the disease for varying periods. Hence, the current notion that chronic patients are synonymous with longer-duration and asymptomatic disease needs re-evaluation. In a survey of patients without classic TED activity (ie, noninflammatory or low CAS), nearly identical percentages of these non-CAS signs and symptoms were seen in longer-duration (mean 9 years) vs shorter-lived (mean 1.4 years) disease, including proptosis, which was present in 59% vs 53%, respectively (3). The current trial examined a unique population of patients with long duration of TED and low CAS who were not previously treated medically.

TED is characterized by overexpression of IGF-1R on cell membranes of orbital fibroblasts and B- and T-cells (21-23), which forms a physical and functional complex with the thyroid-stimulating hormone receptor (TSHR). Activation of this complex by autoantibodies leads to the production of proinflammatory cytokines, adipogenesis in orbital fibroblasts, and overproduction of glycosaminoglycans, like hyaluronan, producing the hallmark signs/symptoms of TED, such as proptosis (24, 25). The overexpression of IGF-1R is not limited to inflammatory disease but is also found in patients lacking the typical inflammatory symptoms of TED, indicating a wide range of utility for targeted therapies beyond patients with active disease (26).

Since the initial successful trials and clinical experience with teprotumumab in reducing the symptoms of moderate to severe, high CAS/active disease, the drug has been reported efficacious in a wide array of patients with TED, including in case series of those with longer disease duration with low and higher CAS (14, 26, 27).

This trial is unique in its placebo-controlled, masked design as well as in its population. It included patients with longer disease duration (mean of 5 years) manifesting proptosis, but with low inflammation (CAS ≤1), who were not candidates for surgery. Arguably, these patients may be more difficult to treat as they may manifest more fibrosis and be irremediable (28).

Patients in this trial had a statistically significant decrease, as assessed by LS mean changes in mm of proptosis and a higher percentage of patients with a 2 mm reduction from baseline (62%) as compared with placebo. The mean observed change in proptosis at Week 24 with teprotumumab from baseline was −2.41 mm, which was smaller than that seen in the largest chronic TED case series published of 31 patients (−3.5 mm) with 90% having a 2 mm reduction at Week 24 (26). Patients in that study had similar beginning proptosis values to this study (24 vs 24.6 mm) with slightly longer mean TED history (approximately 7 vs 5 years) but higher CAS scores (2.3 vs 0.4). They were also older (57 vs 49 years), represented fewer Black patients (13% vs 24%) and fewer former/current smokers (13% vs 41%) than this study. The present study required patients to be stable with regard to disease activity (ie, CAS ≤1) or with no disease progression or new inflammatory symptoms within 1 year of entering the study, and without strabismus or decompression surgery or orbital radiation therapy previously vs the case series where 55% had undergone surgery or radiation before receiving teprotumumab.

Per protocol assessments were consistent with the ITT analyses. Of note, the placebo-assigned patient who received an infusion of teprotumumab in error had a 3 mm improvement in proptosis, and 2 patients in the teprotumumab group missed the Week 18 assessment, possibly contributing to the transient dip in response rate at that time point.

An important feature of this trial design was the inclusion of a placebo control arm, as natural changes in this long-duration, low CAS population are not well documented. Among patients who received placebo, 25% had a proptosis response at Week 24, which was still significantly lower than that observed with teprotumumab (62%).

A reduction in volumes of both retro-orbital muscle and fat was observed in patients on teprotumumab who had MRI, indicating that both compartments are reduced by teprotumumab and contribute to the reduction in proptosis. Previously published studies demonstrated that, over time, there is an increase in orbital fat volume and a decrease in muscle volume, as well as an increase in intramuscular fatty degeneration, in those with longer-duration TED with low disease activity (29). Therefore, it is encouraging to see a mean 35% decrease in fat volumes in those who completed MRI. These findings lead to a conclusion posed by others previously that perhaps teprotumumab can alter neoadipogenesis in the later stages of TED (26). These MRI patients also had large mean improvements from baseline in their overall GO-QOL and appearance-related scores. Visual function–related QOL was near normal at baseline and did not change for these patients, likely because they did not report diplopia at baseline or during the trial.

Positive changes from baseline were noted with the GO-QOL overall (+9), Visual (+8.7) and Appearance (+10) subscales; however, when compared with placebo only the visual subscale reached statistical significance. This lack of significance for the appearance subscale was mostly due to a relatively high appearance-related improvement in the placebo group (+7). This interesting finding deserves further study. Previous data have suggested that patients can adapt to appearance-related changes over time but given the relatively long duration of TED in these patients, this seems unlikely, especially given the relatively poor GO-QOL Appearance subscale scores at baseline (40 in the placebo, and 46.4 in the teprotumumab group). In the only study of GO-QOL in chronic/low inflammation TED known to the authors with a similar duration of TED, appearance-related QOL was also noted to be worse than visually related QOL: Overall GO-QOL score of 60.5 ± 21.8, an appearance subscore of 58.6 ± 24.0, and a visual function subscore of 62.3 ± 25.1, indicating the heavy burden of appearance-related changes in these patients (2). As mentioned earlier, exclusion of past decompression and strabismus surgery from the trial and prohibition of corrective surgeries during the trial could have impacted the appearance-related QOL. The appearance score and improvement can vary widely among individuals, as illustrated by the improved scores of the 6 patients with MRI, including 1 with a 75-point GO-QOL improvement. The GO-QOL data collectively require additional research to further contextualize these findings.

A limitation was that only 14/42 (33%) teprotumumab and 4/20 (20%) placebo patients had diplopia at baseline, making this study underpowered to address potential impact on diplopia. The differences likely relate to a combination of this low power to ascertain benefit and that, by study design, patients were required to have stable disease with no history of prior decompression or strabismus surgery upon study entry. Therefore, patients with chronic diplopia would have had surgery or treatment previously and so would have been ineligible for the study. Another limitation was a short follow-up period (30 days), which did not allow for assessment of improvements over the longer term after completion of treatment.

No new safety signals were identified in this study as compared to previous studies (9, 10). Eighty percent of patients in both the teprotumumab group and the placebo group reported an AE and one patient in each group withdrew due to an AE (hearing dysfunction in teprotumumab patient with congenital ear anomaly and infusion reaction in placebo group). Muscle spasms were reported in 42% of teprotumumab and 10% of placebo patients in this trial. This AE has been noted in previous trials as the most frequent AE and is also listed in the US FDA label for teprotumumab as occurring in 25% of patients (30). Of the AESI, hearing-related events were reported by 22% of teprotumumab patients and 10% of placebo patients and hyperglycemia was reported in 15% of teprotumumab patients vs 10% with placebo, with events managed with medication and no patients discontinuing the study. A recent observational case series reported that 52% had hyperglycemia (31). Differences in study design, including prior steroid use, between the case series and controlled clinical trial may account for some of this variation. The trial required Hb1Ac levels to be ≤8% at baseline, steroid use within 3 weeks was not allowed, and it is not clear if the 2 populations were similar. As stated in the US FDA label, patients should be monitored for these AEs, with appropriate management of hyperglycemia and diabetes, and assessment of hearing impairments before, during. and after treatment (30). There were no events of new onset or exacerbation of inflammatory bowel disease.

## Conclusion

The results from this randomized controlled study add to the increasing body of literature from real-world clinical practice on teprotumumab's efficacy in treating TED. These data demonstrate that teprotumumab significantly reduced proptosis from baseline with a higher rate of proptosis response compared with placebo in patients with longstanding TED and low clinical disease activity. These data taken collectively with previous study results demonstrate teprotumumab's efficacy in TED regardless of disease duration or activity, as indicated in the US FDA label (30). As in previous teprotumumab trials, AESI of hyperglycemia and hearing impairment were noted in this trial. As with any treatment, benefits and risks should be weighed.

## Data Availability

Data will be made available to qualified researchers for independent scientific research. A research proposal and Statistical Analysis Plan must be submitted at medicalinformation@horizontherapeutics.com. Data will be provided following review and approval of the plan and execution of a Data Sharing Agreement. Horizon is committed to responsibly sharing data from the clinical trials we sponsor provided the trials are not part of an ongoing or planned regulatory submission (including requests for clinical trial data for unlicensed products and indications).

## Clinical Trial Information

ClinicalTrials.gov registration no. NCT04583735.

## Abbreviations

AE: adverse event
AESI: adverse event of special interest
CAS: clinical activity score
DKA: diabetic ketoacidosis
FDA: Food and Drug Administration
GO-QOL: Graves’ Ophthalmopathy Quality of Life
HbA1c: glycated hemoglobin
IGF-1: insulin-like growth factor 1
IGF-1R: insulin-like growth factor 1 receptor
ITT: intention-to-treat
mITT: modified intention-to-treat
LS: least squares
MMRM: mixed model for repeated measures
MRI: magnetic resonance imaging
QOL: quality of life
TED: thyroid eye disease
